# Dexmedetomidine Relieves Neuropathic Pain in Rats With Chronic Constriction Injury via the Keap1–Nrf2 Pathway

**DOI:** 10.3389/fcell.2021.714996

**Published:** 2021-09-08

**Authors:** Yatao Liu, Wei Liu, Xiao-Qing Wang, Zhan-Hai Wan, Yong-Qiang Liu, Meng-Jie Zhang

**Affiliations:** ^1^Department of Anesthesiology and Operation, First Hospital of Lanzhou University, Lanzhou, China; ^2^Department of Pathology, Lanzhou University School of Basic Medical Sciences, Lanzhou, China

**Keywords:** chronic constriction injury, neuropathic pain, redox equilibrium, pain behavior, inflammation, apoptosis

## Abstract

This study aimed to determine the role of dexmedetomidine (Dex) in neuropathic pain (NP) after chronic constriction injury (CCI) in a rat model as well as its underlying mechanism. First, a CCI rat model was established. After treatment with Dex, the severity of NP was ascertained by monitoring paw withdrawal threshold (PWT) and paw withdrawal latency (PWL) at different time points. Immunohistochemical analysis was performed to determine the levels of Keap1 and Nrf2 in the spinal cord. Furthermore, the levels of Keap1–Nrf2–HO-1 pathway molecules, apoptotic proteins, and antioxidant genes in the spinal cord or isolated primary microglia were determined using quantitative polymerase chain reaction and western blotting. The release of proinflammatory cytokines was detected via enzyme-linked immunosorbent assay. To evaluate Dex-treated CCI-induced NP via the Keap1–Nrf2–HO-1 pathway, the rats were intrathecally injected with lentivirus to upregulate or downregulate the expression of Keap1. We found that Dex inhibited pathological changes and alleviated sciatic nerve pain as well as repressed inflammation, apoptosis, and redox disorders of the spinal cord in CCI rats. Keap1 protein expression was substantially downregulated, whereas Nrf2 and HO-1 expressions were significantly upregulated in the spinal cord after Dex administration. Additionally, Keap1 overexpression counteracted Dex-mediated inhibition of NP. Keap1 overexpression led to a decrease in Nrf2 and HO-1 levels as well as PWT and PWL but led to an aggravation of inflammation and antioxidant disorders and increased apoptosis. Keap1 silencing alleviated NP in rats with CCI, as evidenced by an increase in PWT and PWL. Keap1 depletion resulted in the alleviation of inflammation and spinal cord tissue injury in CCI rats. Collectively, these findings suggest that Dex inhibits the Keap1–Nrf2–HO-1-related antioxidant response, inflammation, and apoptosis, thereby alleviating NP in CCI rats.

## Introduction

Neuropathic pain (NP), which is chronic pain that arises as a direct consequence of a health condition or a disease damaging the somatosensory system (Popiolek-Barczyk and Mika, 2016), has a complex etiological mechanism and insufficient clinical outcome. Although pathologically, spinal cord neuronal dysfunction is considerably involved in the occurrence of NP, the inhibition of neuronal overactivity has negligible effect in treating the disease. Several studies have linked the important roles played by reactive oxygen species (ROS) production as well as inflammatory changes and central nervous system injury to the pathogenesis of NP (Zimmermann, 2001; Siniscalco et al., 2007; Clark et al., 2013).

Dexmedetomidine (Dex) is a highly selective, sedative, anxiolytic, and analgesic α2-adrenergic agonist with minimal effect on respiration (Cortinez et al., 2004; Hsu et al., 2004). It has widespread application in patients admitted to intensive care units, special populations with respiratory disorders, and pediatric patients (Carollo et al., 2008; Jakob et al., 2014). Dex is used to treat sympathetic storms as well as to prevent the progression of malignant hyperthermia. Previous studies reported that Dex alleviates chronic constriction injury (CCI)-induced NP by mediating the Toll-like receptor 4 (TLR4)–nuclear factor kappa-light-chain-enhancer of activated B cells (NF-kB) (Zhao et al., 2020), mitogen-activated protein kinase (MAPK)–extracellular signal-regulated kinase (ERK) (Lin et al., 2018), and Janus kinase (JAK)–signal transducer and activator of transcription (STAT) (Xun and Zheng, 2020) signaling axes. In the present study, we explored the involvement of the Kelch-like ECH-associated protein 1 (Keap1)–nuclear factor erythroid 2–related factor 2 (Nrf2) pathway in Dex-ameliorated NP.

A number of regulatory factors have been found to play important roles in maintaining oxidant homeostasis in cells during intracellular antioxidant defense processes. The transcription factor Nrf2 plays a crucial role in the regulation of glutathione S-transferase and NAD(P)H quinone dehydrogenase 1 (NQO1), among other antioxidant enzymes, by binding to antioxidant response elements (AREs), which are DNA enhancer sequences (Zhang, 2010; Ma and He, 2012). The poor prognosis of patients with hepatocellular carcinoma, lung cancer, bladder cancer, or several other cancers is associated with the stability and activation of Nrf2 (Ma and He, 2012). Nrf2 binds to the Cul3–Rbx1–Keap1 ligase complex in the cytoplasm and severely represses its expression and activity, thereby limiting its translocation from the cytoplasm to the nucleus. Subsequently, constitutive Nrf2 expression maintains basal antioxidant levels (Ma and He, 2012). Activation of the Keap1–Nrf2 pathway has been shown to promote the release of interleukin (IL)-1β, IL-6, and tumor necrosis factor-α (TNF-α) as well as neuronal apoptotic cell death and superoxide production (Satoh et al., 2006, 2009; Li et al., 2018). This results in an increase in the development or persistence of pain. In the present study, we explored the involvement of the Keap1–Nrf2 axis in Dex-treated CCI-induced NP. We demonstrated that Dex considerably represses mechanical antipain signaling and thermal hyperalgesia as well as downregulates the levels of apoptotic markers and inflammatory cytokines but promotes antioxidant gene expression. Moreover, we demonstrated that Dex activates CCI-inhibited signal transduction via the Keap1–Nrf2 pathway.

## Materials and Methods

### Ethics

All experimental procedures were performed in strict accordance with the requirements established by the National Institutes of Health for the Care and Use of Laboratory Animals after approval from the Ethics Committee of The First Hospital of Lanzhou University on June 22, 2017.

### Animals

Adult male Sprague Dawley rats weighing 190–210 g were acquired from Beijing Life River Experimental Animal Center. They were housed in cages, with eight rats per cage, at a constant temperature of 20–22°C and a 12-h/12-h light/dark cycle with *ad libidum* access to food and water. The feeding behavior, daily activities, and weight changes of the rats were closely monitored and recorded daily. After 7 days of acclimatization, the rats were randomized into two groups: one group underwent CCI treatment and the other underwent sham operation.

### Induction of Neuropathic Pain

Before surgery, every precaution was taken to prevent animal suffering, especially to the sciatic nerve. The rats (180–220 g) were housed at a constant room temperature of 25°C under a 12-h/12-h light/dark cycle and had *ad libidum* access to food and water. The rats were randomly assigned to the sham group (*n* = 8) or CCI group (*n* = 32). NP was surgically induced by CCI surgery following a previously described procedure (Bennett and Xie, 1988). Before surgery, precaution was taken to avoid damage to the sciatic nerve. The rats were anesthetized with pentobarbital (60 mg/kg, i.p.), and the left sciatic nerve was exposed to separate it from the underlying connective tissue. The nerves were ligated three times using 4-0 chromic gut sutures. Typical hind paw twitching occurred when the nerves were contracted. The same silk thread was then used to suture the overlying muscle and skin. The sham group was subjected to the same procedure but without sciatic nerve ligation. The sutures were not tied too tightly, and blood flow was barely constricted. All rats were administered antibiotics (penicillin, 0.5 ml, 96 mg/ml, s.c.) to lower the possibility of infection. The operations were performed by an investigator skilled in modeling to minimize variability.

### Behavioral Examination

Paw withdrawal threshold (PWT) and paw withdrawal latency (PWL) were measured to assess mechanical allodynia and thermal hyperalgesia, respectively. To assess mechanical allodynia, all rats were placed in transparent plastic chambers with metal mesh floors, and the surface of the plantar paw of each rat was stimulated by calibrated von Frey filaments (Dynamic Plantar Esthesiometer; Ugo Basile, Milan, Italy) based on a previously described method (Cunha et al., 2004). Five trials were performed, and the weakest force (g) that resulted in paw withdrawal at least three times was recorded as PWT.

Paw withdrawal latency was measured by placing each rat on a glass panel with a radiant heat generator beneath the panel at a preset temperature of 54 ± 0.1°C (Hargreaves et al., 1988). PWL was measured twice, 5 min apart, and the average value was considered. In case of paw withdrawal under pressure, the time was recorded. Both trials were conducted by investigators blinded to the experiments.

### Dex Treatment

Anesthesia was induced by pentobarbital (60 mg/kg i.p.). Then, 16 rats were injected with 15 g/kg Dex i.p. at 5 g/kg/h for 3 h prior to CCI surgery based on the method described by Zhang et al. (2020); the remaining 16 rats were not treated with Dex.

### Production of Lentiviral Vectors and Intrathecal Injection

According to the manufacturer’s instructions, lentiviral vectors LV-shRNA-Keap1, LV-shRNA-NC, LV-Keap1, and LV-NC were constructed. Rats were anesthetized with sodium pentobarbital (60 mg/kg, i.p.). A PE-10 polyethylene catheter (cut into an approximately 15-cm segment) was then inserted into the subarachnoid space according to a previously described method (Sakura et al., 1996) for intrathecal implantation. The catheters were passed through a slit in the atlanto-occipital membrane and advanced 11 cm so that the tip was caudal to the conus medullaris. The rats were allowed to recover for 24 h before the study began. Rats that exhibited any evidence of sensory or motor dysfunction were excluded from the study. The catheter was injected with 10 μl of recombinant lentivirus (GenePharma) via a microinjection syringe for intrathecal treatment. Lidocaine (5 mg/kg s.c., Sigma) was injected to paralyze the bilateral hind limbs to determine the functionality and position of the catheter tip in the subarachnoid space.

### Enzyme-Linked Immunosorbent Assay

Enzyme-linked immunosorbent assay (ELISA) was performed according to a previous description (You et al., 2019). Tissue samples from the dorsal horn of the spinal cord were suspended in a mixture of lysis buffer and protease inhibitor cocktail (Roche Applied Science) prior to incubation on ice for 5 min. Then, the samples were centrifuged at 10,000 × *g* at 4°C for 10 min, and the supernatant was collected. The levels of inflammatory factors TNF-α, IL-6, and IL-8 were determined using an ELISA kit according to the manufacturer’s instructions. The optical density was measured at a wavelength of 450 nm using the Bio-Rad ELISA reader.

### Quantitative Polymerase Chain Reaction

The mRNA levels of Keap1, Nrf2, HO-1, caspase-9, Bax, and Bak were measured using quantitative polymerase chain reaction (qPCR). qPCR were performed as described in a previous study (Wang et al., 2018). On the 21st day after CCI surgery, once the rats had been decapitated, total RNA was extracted from L4–L5 spinal cord tissue using the RNAiso Plus kit (Takara Biotechnology, Dalian, China) according to the manufacturer’s instructions. The isolated RNA was reverse-transcribed, and the first strand of cDNA was synthesized using an oligonucleotide dT18 primer. A cDNA synthesis kit (Takara Biotechnology) was used as a template for PCR amplification using the SYBR Premix Ex Taq^TM^ II kit (Takara Biotechnology). The mRNA levels of actin were used as an internal reference.

### Western Blotting

Western blotting (WB) was performed according to a previous study (Li et al., 2016). Protein samples were subjected to electrophoretic separation and transferred onto a polyvinylidene fluoride membrane (Bio-Rad). Then, the proteins were incubated with 2.5% relative blocking buffer according to standard procedures. The membranes were then incubated with the following primary antibodies: anti-Keap1 (ab119403), anti-Nrf2 (ab31163), anti-HO-1 (ab13243), anti-Caspase-9 (ab25758), anti-Bax (ab53154), anti-Bak (ab69404), and anti-GAPDH (ab8245). NIH/ImageJ software (version 1.8.0_45, Amersham, United Kingdom) was used to quantify the protein bands.

### Antioxidant Assays

The GSH-Glo^TM^ assay kit (Promega) (Scherer et al., 2008), catalase (CAT) assay kit (BioVision) (Erdem et al., 2020), superoxide dismutase (SOD) assay kit (Sigma-Aldrich) (Erdem et al., 2020), and total antioxidant capacity (TAC) assay kit (Beyotime Biotechnology) were used to measure glutathione (GSH) content, CAT activity, SOD activity, and TAC, respectively.

### Luciferase Activity

The luciferase reporter assay was performed to determine the ARE activity (Westerink et al., 2010). First, AREs were cloned into the pGL3-Basic reporter plasmid. The isolated microglial cells were seeded in triplicate at densities of 1 × 10^5^ cells/well into a 12-well plate and incubated for 24 h. Two hundred nanograms of ARE luciferase reporter plasmid was added to transfect the cells using the OriGene MegaTran 1.0 transfection reagent according to the manufacturer’s instructions. The cells were then allowed to recover for 24 h in a medium containing 10% fetal bovine serum. At 48 h after transfection, firefly and nephridium signals were measured using the Promega Dual Luciferase Reporter Assay kit, which showed increased activation compared with the reporter alone.

### Immunohistochemistry

Tissue samples were extracted from the bottom of the dorsal spinal cord at 4°C by immersing it in 4% formaldehyde solution overnight, followed by paraffin embedding. The samples were sliced into 4-μm-thick sections, dewaxed with xylene, and rehydrated with serial ethanol solutions. Next, the sections were recovered with antigen by soaking them in 0.01 M citrate buffer (pH 6.0) and then heating them in a microwave oven for 30 min. Their endogenous peroxidase activity was blocked with 0.3% hydrogen peroxide. The sections were then incubated with primary antibodies overnight at 4°C and then with secondary antibodies at 37°C for 1 h. After rinsing the sections, they were treated with diaminobenzidine (He et al., 2012). Finally, the cells were observed under a microscope (×200).

### Statistical Analyses

Data were presented as mean ± SEM. Significance between two groups and among multiple groups was determined using Student’s *t*-test or one way analysis of variance with Tukey’s *post hoc* test. *P* values of <0.05 were considered statistically significant.

## Results

### Effect of Dex Treatment on Pain, Inflammation, and Apoptosis

**FIGURE 1:**
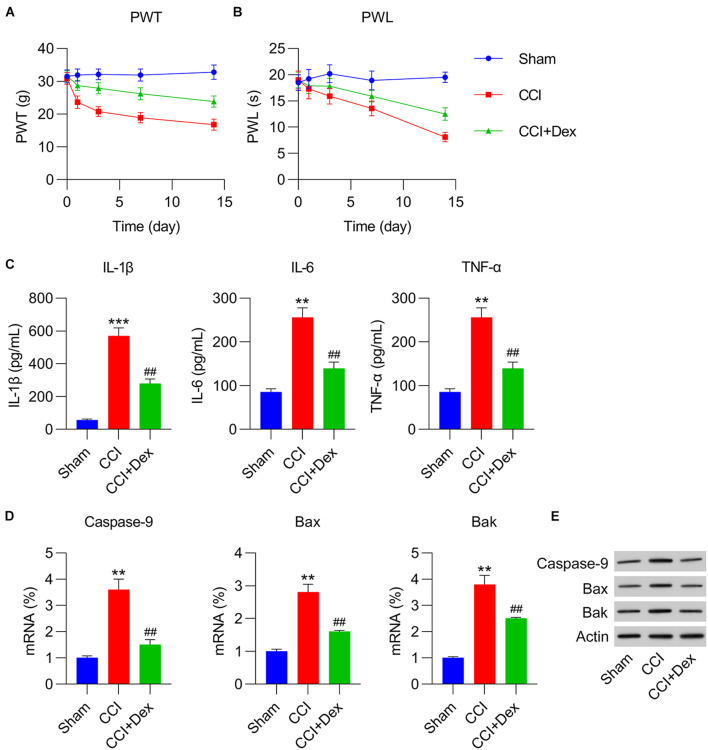
Dexmedetomidine (Dex) mitigated chronic constriction injury (CCI)-induced neuropathic pain. **(A)** Paw withdrawal threshold (PWT) (g) at 0, 1, 3, 7, and 14 days after operation. **(B)** Paw withdrawal latency (PWL) (s) at 0, 1, 3, 7, and 14 days after operation. **(C)** IL-1β, IL-6, and TNF-α expression levels were determined using enzyme-linked immunosorbent assay. **(D)** mRNA levels of caspase-9, Bax, and Bak in spinal cord tissues were determined using quantitative polymerase chain reaction. **(E)** Protein levels of caspase-9, Bax, and Bak in spinal cord tissues were determined using western blotting. **P < 0.01, ***P < 0.001 vs. Sham group; ^##^*P* < 0.01 vs. CCI group.

Following surgery, CCI rats were injected with Dex to determine its role in the development of NP. According to the data, Dex significantly abated thermal hyperalgesia and mechanical allodynia (Figures 1A,B). Because inflammatory cytokines and apoptosis play an essential role in NP irritation, the levels of IL-1β, IL-6, and TNF-α were measured using ELISA. The results showed that these three inflammatory factors were robustly produced in the CCI group, whereas Dex treatment obviously inhibited their expression (Figure 1C). qPCR and WB were performed to determine the expressions of the pro-apoptotic factors caspase-9, Bax, and Bak. The expressions of caspase-9, Bax, and Bak increased in the CCI group compared with those in the sham group; Dex treatment substantially ameliorated the expressions of these markers (Figures 1D,E).

### Effect of Dex Treatment on Antioxidant Response and Keap1–Nrf2 Activation

**FIGURE 2:**
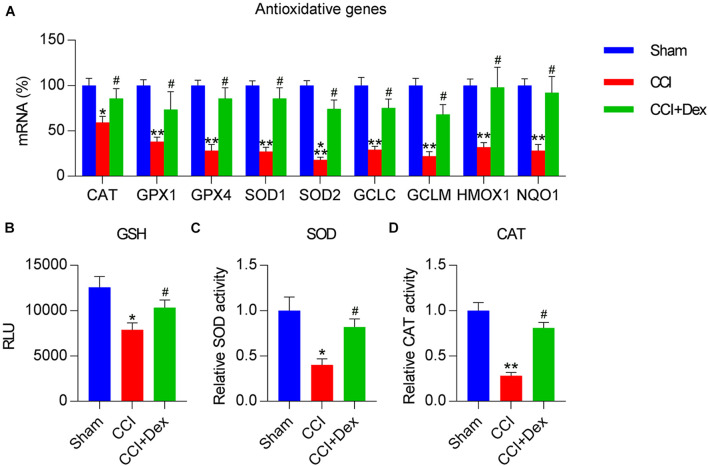
Dexmedetomidine promoted antioxidant capacity in CCI-induced rats. **(A)** Expression levels of antioxidant genes were determined using quantitative polymerase chain reaction. **(B)** Analysis of glutathione (GSH) levels. **(C)** A superoxide dismutase (SOD) assay kit was used to determine SOD activity. **(D)** Determination of catalase (CAT) activity in spinal cord tissues. *P < 0.05, **P < 0.01 vs. Sham group; ^#^*P* < 0.05 vs. CCI group.

Because decreased antioxidant response is a manifestation of NP and nerve injury (Tan et al., 2009; Kallenborn-Gerhardt et al., 2013), we determined the expressions of antioxidant genes in spinal cord tissues of the rats using qPCR. The expressions of antioxidant genes, including *CAT*, *GPX1*, *GPX4*, *SOD1*, *SOD2*, glutamate-cysteine ligase (*GCL*), *GCLC*, *GCLM*, *HMOX-1*, and *NQO1* (Wang et al., 2019), were downregulated in the CCI group compared with those in the sham group. Furthermore, Dex treatment counteracted the effect of CCI surgery on the levels of these genes (Figure 2A). In addition, CCI resulted in the downregulation of antioxidant responses, including GSH and SOD/CAT activities, all of which were restored in CCI rats following Dex treatment (Figures 2B–D).

### Dex Treatment Reduced Keap1 Expression but Upregulated Nrf2 Expression

**FIGURE 3:**
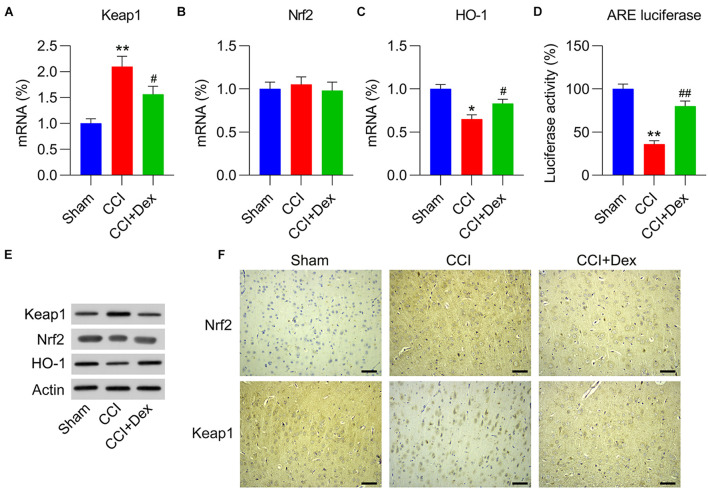
Role of Dex in Keap1–Nrf2 pathway expression and activation in CCI-induced rats. **(A,B)** mRNA expression levels of Keap1 and Nrf2 were measured using quantitative polymerase chain reaction (qPCR). **(C)** mRNA expression levels of HO-1 were measured using qPCR. **(D)** Isolated microglia were transfected with ARE luciferase vector, followed by determination of luciferase activity in the cells at 36 h after transfection. **(E)** Protein expression levels of Nrf2, Keap1, and HO-1 were measured using western blotting. **(F)** Immunohistochemical staining of Keap1 and Nrf2 expression in spinal cord tissues. Scale bar, 50 μm. *P < 0.05, **P < 0.01 vs. Sham group; ^#^*P* < 0.05, ^##^*P* < 0.01 vs. CCI group.

To determine whether the Keap1–Nrf2 axis, a well-recognized antioxidant pathway, participates in Dex-ameliorated NP in CCI rats, the mRNA and protein expression levels of Keap1 and Keap1-destablized Nrf2 were determined using qPCR and WB, respectively. The results clearly demonstrated that Keap1 expression in the spinal cord was upregulated by CCI surgery, whereas Dex treatment reversed the effect of CCI surgery on Keap1 expression (Figures 3A,E). However, qPCR showed that there were no differences in Nrf2 expression among the sham, CCI, and CCI + Dex groups. In contrast, WB showed that Nrf2 protein level were downregulated due to CCI surgery, whereas Dex treatment increased Nrf2 protein levels in the spinal cord of rats (Figures 3B,E). We next determined Nrf2-downstream HO-1 expression and activation of AREs. qPCR and WB results showed that CCI surgery downregulated HO-1 expression but upregulated HO-1 levels in the Dex-administered group (Figures 3C,E). In addition, ARE luciferase activity was remarkably decreased in CCI rats; however, Dex treatment resulted in the upregulation of ARE luciferase activity compared with that in the CCI group (Figure 3D). IHC staining results also indicated that Nrf2 expression was upregulated and Keap1 expression was downregulated in the CCI group; while Dex treatment reversed their expressions in the nervous tissue (Figure 3F). Collectively, these data suggest that CCI surgery blocks the signal transduction of the Keap1–Nrf2 pathway by upregulating Keap1 expression, whereas Dex treatment downregulates Keap1 expression, thereby activating Nrf2 and downstream sensors.

### Involvement of the Activated Keap1–Nrf2 Pathway in Dex-Alleviated NP

**FIGURE 4:**
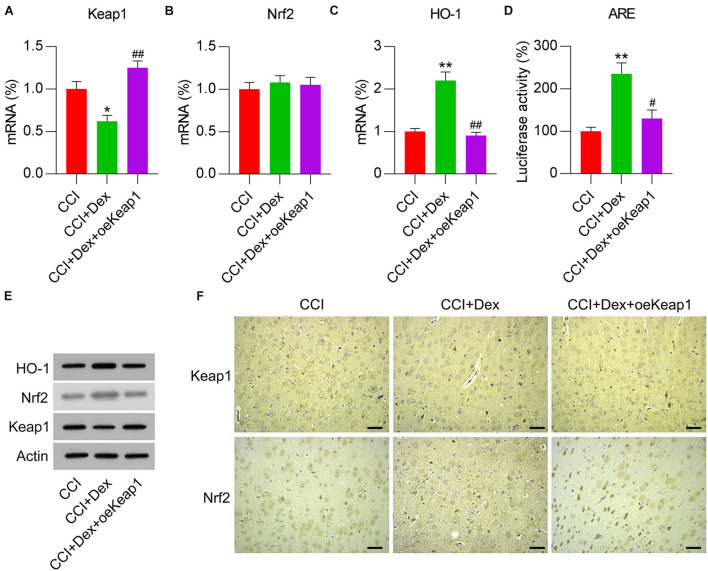
Overexpression of Keap1 in Dex-treated CCI-induced rats intrathecally injected with lentiviral-NC or lentiviral-Keap1 for 1 week. Rats were then subjected to Dex treatment and CCI surgery. **(A,B)** mRNA expression levels of Keap1 and Nrf2 was measured using qPCR. **(C)** mRNA expression level of HO-1 in the spinal cord was detected using qPCR. **(D)** Isolated microglia were transfected with ARE luciferase vectors, and luciferase activity was determined in the cells at 36 h after transfection. **(E)** Protein expression levels of Nrf2, Keap1, and HO-1 were measured using WB. **(F)** Immunohistochemical staining of Keap1 and Nrf2 expression in spinal cord tissues. Scale bar, 50 μm. *P < 0.05, **P < 0.01 vs. CCI group; ^#^*P* < 0.05, ^##^*P* < 0.01 vs. CCI+Dex group.

To elucidate the effect of the Keap1–Nrf2 pathway on Dex-alleviated NP in CCI rats, Keap1 expression was upregulated by intrathecally injecting lentiviral controls or Keap1 into Dex-administered rats. qPCR revealed that lentiviral-Keap1 injection contributed toward Keap1 overexpression in CCI + Dex rats compared with that in the CCI + Dex group (Figures 4A,E). The Nrf2 mRNA levels showed no difference between the CCI + Dex group and CCI + Dex + oeKeap1 and CCI + Dex groups; however, the Nrf2 protein levels decreased in the spinal cord after lentiviral-Keap1 injection (Figures 4B,E). In addition, the HO-1 mRNA and protein levels decreased due to Keap1 upregulation (Figures 4C,E). Furthermore, ARE luciferase activity was significantly decreased after Keap1 overexpression (Figure 4D). IHC staining results also showed that Keap1 overexpression downregulated Nrf2 expression in neural tissues (Figure 4F). These data demonstrate that lentiviral-Keap1 injection contributes toward the attenuated activation of the Keap1–Nrf2 pathway.

**FIGURE 5:**
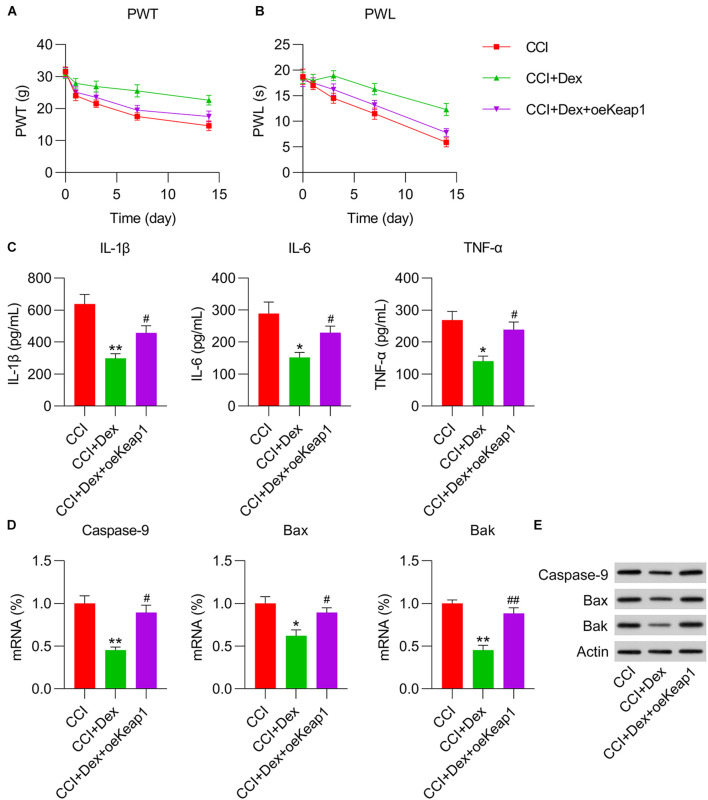
Effect of Keap1 overexpression on Dex-alleviated neuropathic pain in CCI-induced rats intrathecally injected with lentiviral-NC/lentiviral-Keap1 for 1 week. Rats were then subjected to Dex treatment and CCI surgery. **(A)** Paw withdrawal threshold (g) at 0, 1, 3, 7, and 14 days after operation. **(B)** Paw withdrawal latency (s) at 0, 1, 3, 7, and 14 days after operation. **(C)** IL-1β, IL-6, and TNF-α expression levels were measured in spinal cord tissues using ELISA. **(D)** mRNA expression levels of caspase-9, Bax, and Bak were determined in spinal cord tissues using quantitative polymerase chain reaction. **(E)** Protein expression levels of caspase-9, Bax, and Bak in spinal cord tissues were determined using western blotting. *P < 0.05, **P < 0.01 vs. CCI group; ^#^*P* < 0.05, ^##^*P* < 0.01 vs. CCI+Dex group.

To determine the effect of Keap1 overexpression on neuralgia in CCI rats, we measured PWT and PWL. Both PWT and PWL were remarkably higher in Dex-treated CCI rats than in the CCI group. However, Keap1 upregulation significantly increased PWT and PWL compared with those in the CCI + DEX group (Figures 5A,B). This result suggests that Keap1 overexpression promotes neuralgia in CCI rats.

To determine the role of Keap1 upregulation on inflammation and apoptotic changes in the spinal cord tissue of CCI rats, ELISA, qPCR, and WB were performed to measure the levels of inflammatory cytokines and proapoptotic factors. ELISA results revealed that Keap1 restored inflammatory cytokine production in Dex-treated CCI rats (Figure 5C). qPCR and WB revealed that the mRNA and protein expressions of caspase-9, Bax, and Bak were significantly elevated in Dex-treated CCI rats with Keap1 upregulation than in CCI + Dex rats (Figures 5D,E).

**FIGURE 6:**
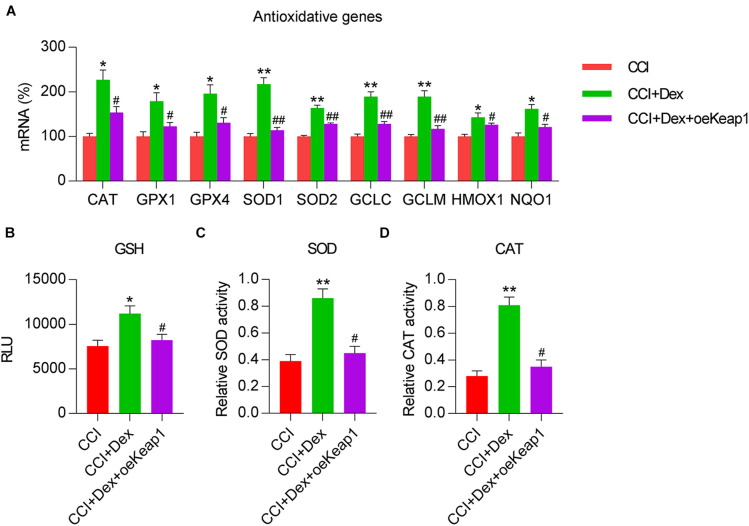
Effect of Keap1 overexpression on Dex-alleviated antioxidant capacity in spinal cord tissues of rats. Rats were intrathecally injected with lentiviral-NC or lentiviral-Keap1 for 1 week and then subjected to Dex treatment and CCI surgery. **(A)** Levels of antioxidant genes in the spinal cord were determined using quantitative polymerase chain reaction. **(B)** Analysis of glutathione levels in spinal cord tissue. **(C)** Superoxide dismutase activity in spinal cord tissues was examined using a SOD assay kit. **(D)** Analysis of catalase activity. *P < 0.05, **P < 0.01 vs. CCI group; ^#^*P* < 0.05, ^##^*P* < 0.01 vs. CCI+Dex group.

We then investigated whether Keap1 overexpression mitigated the effects of Dex treatment on the upregulation of antioxidant gene expression. qPCR revealed that the expression levels of *GPX4*, *SOD1*, *SOD2*, *HMOX1*, *NQO1*, *CAT*, *GPX1*, *GCLC*, and *GCLM* were significantly decreased in the spinal cord of CCI rats after Keap1 overexpression (Figure 6A). Additionally, the activities of three antioxidants, i.e., GSH, SOD, and CAT, were downregulated following Keap1 overexpression compared with their activities in the CCI + Dex group (Figures 6B–D). Collectively, these data suggest that Keap1 is involved in Dex-regulated redox equilibrium in the spinal cord of rats.

### Keap1 Silencing Alleviated NP

**FIGURE 7:**
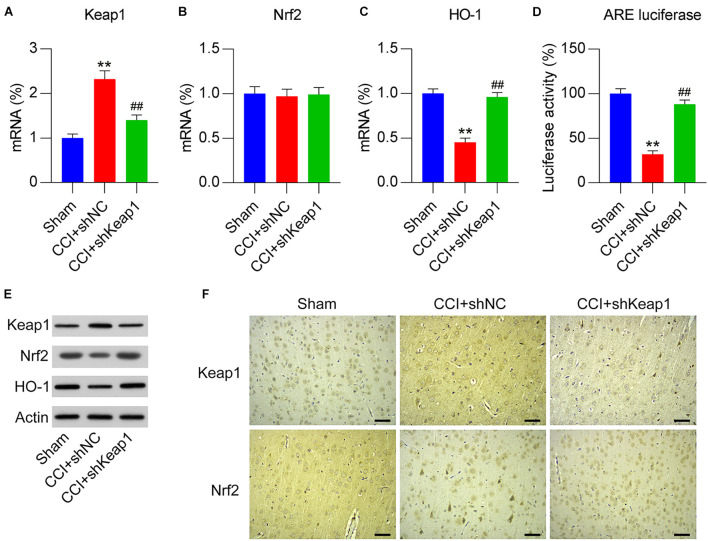
Keap1 knockdown in CCI-induced rats intrathecally injected with lentiviral-shNC or lentiviral-shKeap1 for 1 week. Rats were then subjected to CCI surgery. **(A,B)** Expression levels of Keap1 and Nrf2 in the spinal cord were measured using qPCR. **(C)** mRNA expression level of HO-1 in the spinal cord was assayed using qPCR. **(D)** Isolated microglia were transfected with ARE luciferase vector, and luciferase activity was measured at 36 h after transfection. **(E)** Protein expression levels of Keap1, Nrf2, and HO-1 were determined using western blotting. **(F)** Immunohistochemical staining for Keap1 and Nrf2 expression. Scale bar, 50 μm. **P < 0.01 vs. Sham group; ^##^*P* < 0.01 vs. CCI+shNC group.

To characterize the direct involvement of the Keap1–Nrf2 pathway in NP in CCI rats, Keap1 knockout was performed in CCI rats by intrathecally injecting lentiviral-shKeap1. qPCR and WB results revealed that compared with those in the CCI group, Keap1 mRNA and protein levels were significantly decreased (Figures 7A,E). However, Nrf2 expression was only elevated at the protein level due to Keap1 depletion (Figures 7B,E). Furthermore, HO-1 mRNA and protein levels were obviously downregulated, whereas ARE luciferase activity was also reduced due to Keap1 silencing (Figures 7C,E). ARE luciferase activity was significantly elevated after Keap1 silencing (Figure 7D). IHC staining results confirmed that Nrf2 levels in neural tissues were promoted by Keap1 depletion (Figure 7F).

**FIGURE 8:**
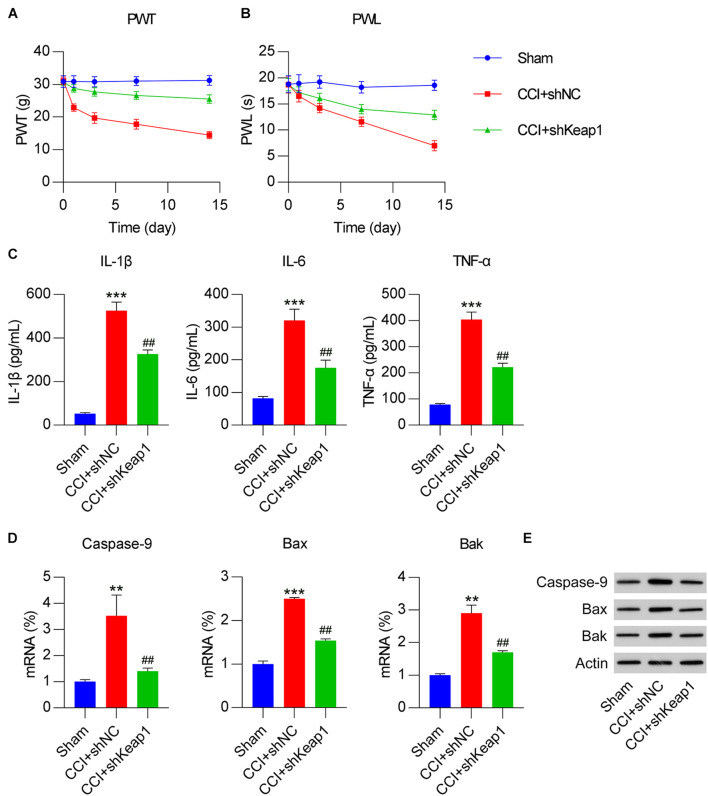
Effect of Keap1 overexpression on Dex-alleviated neuropathic pain in CCI-induced rats intrathecally injected with lentiviral-shNC/lentiviral-shKeap1 for 1 week. Rats were then subjected to CCI surgery. **(A)** Paw withdrawal threshold (g) at 0, 1, 3, 7, and 14 days after operation. **(B)** Paw withdrawal latency (s) at 0, 1, 3, 7, and 14 days after operation. **(C)** Expression levels of IL-1β, IL-6, and TNF-α was identified in the spinal cord using enzyme-linked immunosorbent assay. **(D)** mRNA expression levels of apoptotic factors caspase-9, Bax, and Bak in spinal cord tissues were determined using quantitative polymerase chain reaction. **(E)** Protein expression levels of caspase-9, Bax, and Bak in spinal cord tissues were determined using western blotting. **P < 0.01, ***P < 0.001 vs. Sham group; ^##^*P* < 0.01 vs. CCI+shNC group.

Paw withdrawal threshold and PWL were measured in sham, CCI, and CCI + shKeap1 rats after surgery. Mechanical allodynia (Figure 8A) and thermal hyperalgesia (Figure 8B) were improved in Keap1-silenced rats compared with those in CCI rats. Moreover, the levels of proinflammatory cytokines in the spinal cord induced by CCI surgery were repressed by Keap1 silencing (Figure 8C). In addition, the levels of caspase-9, Bax, and Bak were obviously decreased in Keap1-silenced rats compared with those in CCI rats (Figures 8D,E).

**FIGURE 9:**
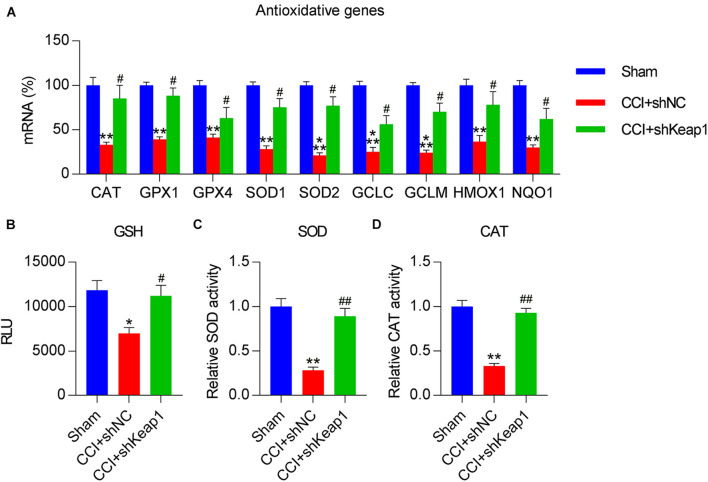
Effect of Keap1 silencing on CCI-repressed antioxidant capacity in spinal cord tissues of rats. Rats were intrathecally injected with lentiviral-shNC or lentiviral-shKeap1 for 1 week and then subjected to CCI surgery. **(A)** Levels of antioxidant genes in the spinal cord were determined via quantitative polymerase chain reaction. **(B)** Analysis of glutathione levels. **(C)** A superoxide dismutase assay kit was used to examine SOD activity. **(D)** Determination of catalase activity. *P < 0.05, **P < 0.01 vs. Sham group; ^#^*P* < 0.05, ^##^*P* < 0.01 vs. CCI+shNC group.

Finally, we determined the levels of antioxidant genes in CCI rats after Keap1 silencing to ascertain the direct role of Keap1 in redox equilibrium. qPCR results showed that antioxidant gene expressions were clearly promoted in Keap1-silenced rats than in CCI rats (Figure 9A). Moreover, GSH levels and SOD and CAT activities were upregulated after Keap1 silencing (Figures 9B–D). Taken together, these data suggest that CCI-induced inflammation, apoptosis, and oxidant disorders are repressed by Keap1 silencing.

## Discussion

In this study, we subjected rats with CCI-induced NP to Dex treatment and revealed the protective role of the procaine against NP. Dex-treated rats were less sensitive to mechanical and thermal stimulation and showed high activation of the Keap1–Nrf2 signaling axis compared with CCI rats. Keap1 overexpression partially counteracted the effects of Dex on NP behaviors as well as neural inflammation and apoptosis; in contrast, Keap1 silencing showed similar effects as that of Dex treatment following CCI surgery. These data suggest that Dex ameliorates CCI-induced NP via the activation of the Keap1–Nrf2 pathway.

The ectopic upregulation of Nrf2 has been reported in multiple diseases, particularly cancers. Clinically, patients with high Nrf2 levels usually have poor prognosis (Liew et al., 2015; Lu et al., 2017). High Nrf2 expression is associated with antioxidant responses and metabolic reprogramming. Nrf2 reprograms glucose and glutamine metabolism to redirect to anabolic pathways, thereby facilitating uncontrolled cancer cell proliferation (Mitsuishi et al., 2012). Furthermore, it functions in the hypoxia-inducible factor-1α pathway to regulate aerobic glycolysis (Zhang et al., 2018). Recently, HO-1 expression induced by NRF2 has been increasingly demonstrated to exert a promising analgesic effect. According to Shen et al. (2015), the intraperitoneal injection of the HO-1 inducer CoPP and administration of lentivirus expressing HO-1 via intraspinal injection mitigated vincristine-induced neuralgia. CoPP has a similar analgesic effect, as demonstrated in a mouse model with NP stimulated via L5 spinal cord ligation (Liu et al., 2016). However, analgesia mediated by sulforaphane, an Nrf2 activator, could be fully blocked by protoporphyrin, a HO-1 inhibitor, according to Kim D. et al. (2010). Zhou et al. (2020a) demonstrated that oltipraz activates the Nrf2–HO-1 signaling pathway and may attenuate paclitaxel-induced neuralgia. PPARγ activation was shown to induce the Nrf2–HO-1 signaling pathway, thereby alleviating mechanical allodynia in paclitaxel-induced neuralgia (Zhou et al., 2020b). These findings indicate that the Keap1/Nrf2 signaling axis is critically associated with the analgesic effect during neuralgia. In the present study, Dex treatment contributed toward the activation of the Keap1–Nrf2 axis, as evidenced by upregulated HO-1 expression and ARE luciferase activity. Keap1 overexpression in Dex-treated CCI rats abolished Dex inhibition of NP, whereas Keap1 silencing in CCI rats alleviated NP, confirming the suppressive role of Nrf2 in NP in rats.

Previous studies reported that ROS scavengers considerably decrease the progression of NP in animal models (Kim H. K. et al., 2010; Fidanboylu et al., 2011; Kim et al., 2016). A number of antioxidants have been clinically shown to protect against NP (Argyriou et al., 2006; Ghoreishi et al., 2012), suggesting that it is important to maintain cellular redox equilibrium to prevent NP. The present study results indicated that CCI surgery in rats resulted in a significant decrease in the expression and activity of antioxidant genes in spinal cord tissue; however, this decrease could be partially recovered by Dex treatment. Loss and gain experiments revealed that Keap1 overexpression counteracts the effect of Dex treatment on antioxidant gene expression, whereas Keap1 silencing has a role similar to that of Dex treatment. These data suggest that Dex plays a role in maintaining redox homeostasis by regulating Keap1 levels in CCI rats.

Many studies suggested that Nrf2 activation was related to inflammatory responses (Innamorato et al., 2008) and levels of apoptotic factors (Abdalkader et al., 2018). Nrf2 is considered a crucial modulator of inflammation and apoptosis in various conditions, including cancers, and regulates endogenous defenses against multiple cellular stresses, particularly oxidative stress (Sajadimajd and Khazaei, 2018). The Nrf2 pathway is also widely expressed in the central nervous system and is regulated in response to both acute cerebral insults and in neurodegenerative diseases (Hubbs et al., 2007; Rojo et al., 2010). Although Keap1–Nrf2 pathway is well documented in NP in previous studies (Li et al., 2018; Kalvala et al., 2020), its association with Dex was not reported, which is considered as one of innovation of this study. In the present study, inflammation and apoptosis levels were determined in the spinal cord tissues of Dex-treated CCI rats by measuring the levels of cytokines and apoptotic factors using ELISA, qPCR, and WB. Compared with sham rats, CCI rats showed a higher level of inflammation and apoptosis; this result is consistent with that of a previous study (Zhang et al., 2020); in contrast, Dex treatment decreased the overexpression of inflammatory cytokines and apoptotic markers. We further found that the activated Keap1–Nrf2 pathway, as indicated via downstream HO-1 expression and ARE luciferase activity, is inversely associated with the generation of inflammation and apoptotic changes in CCI rats.

In summary, Dex mitigated mechanical allodynia, excessive cytokine production, thermal hyperalgesia, apoptotic factor production, and redox disorders in a rat model of NP but promotes the activation of the Keap1–Nrf2 signaling axis. Our findings reveal a novel mechanism of Dex-ameliorated neuralgia.

## Data Availability Statement

The original contributions presented in the study are included in the article/supplementary material, further inquiries can be directed to the corresponding author.

## Ethics Statement

All trial procedures were performed in strict accordance with the requirements established by the National Institutes of Health for the Care and Use of Laboratory Animals after approval from the Ethics Committee at The First Hospital of Lanzhou University on 22 June 2017.

## Author Contributions

YL, WL, X-QW, and Z-HW conceived the project and designed and performed the experiments. YL, WL, X-QW, Z-HW, Y-QL, and M-JZ analyzed the data. YL wrote and revised the manuscript. All authors contributed to the article and approved the submitted version.

## Conflict of Interest

The authors declare that the research was conducted in the absence of any commercial or financial relationships that could be construed as a potential conflict of interest.

## Publisher’s Note

All claims expressed in this article are solely those of the authors and do not necessarily represent those of their affiliated organizations, or those of the publisher, the editors and the reviewers. Any product that may be evaluated in this article, or claim that may be made by its manufacturer, is not guaranteed or endorsed by the publisher.
